# A Novel Virtual Reality Medical Image Display System for Group Discussions of Congenital Heart Disease: Development and Usability Testing

**DOI:** 10.2196/20633

**Published:** 2020-12-08

**Authors:** Byeol Kim, Yue-Hin Loke, Paige Mass, Matthew R Irwin, Conrad Capeland, Laura Olivieri, Axel Krieger

**Affiliations:** 1 University of Maryland College Park, MD United States; 2 Children's National Hospital Washington, DC United States; 3 Indicated LLC New York City, NY United States

**Keywords:** virtual reality, cardiac diagnostics, usability study, congenital heart disease, group collaboration

## Abstract

**Background:**

The complex 3-dimensional (3D) nature of anatomical abnormalities in congenital heart disease (CHD) necessitates multidisciplinary group discussions centered around the review of medical images such as magnetic resonance imaging. Currently, group viewings of medical images are constrained to 2-dimensional (2D) cross-sectional displays of 3D scans. However, 2D display methods could introduce additional challenges since they require physicians to accurately reconstruct the images mentally into 3D anatomies for diagnosis, staging, and planning of surgery or other therapies. Virtual reality (VR) software may enhance diagnosis and care of CHD via 3D visualization of medical images. Yet, present-day VR developments for medicine lack the emphasis on multiuser collaborative environments, and the effect of displays and level of immersion for diagnosing CHDs have not been studied.

**Objective:**

The objective of the study was to evaluate and compare the diagnostic accuracies and preferences of various display systems, including the conventional 2D display and a novel group VR software, in group discussions of CHD.

**Methods:**

A total of 22 medical trainees consisting of 1 first-year, 10 second-year, 4 third-year, and 1 fourth-year residents and 6 medical students, who volunteered for the study, were formed into groups of 4 to 5 participants. Each group discussed three diagnostic cases of CHD with varying structural complexity using conventional 2D display and group VR software. A group VR software, Cardiac Review 3D, was developed by our team using the Unity engine. By using different display hardware, VR was classified into nonimmersive and full-immersive settings. The discussion time, diagnostic accuracy score, and peer assessment were collected to capture the group and individual diagnostic performances. The diagnostic accuracies for each participant were scored by two experienced cardiologists following a predetermined answer rubric. At the end of the study, all participants were provided a survey to rank their preferences of the display systems for performing group medical discussions.

**Results:**

Diagnostic accuracies were highest when groups used the full-immersive VR compared with the conventional and nonimmersive VR (χ^2^_2_=9.0, *P*=.01) displays. Differences between the display systems were more prominent with increasing case complexity (χ^2^_2_=14.1, *P*<.001) where full-immersive VR had accuracy scores that were 54.49% and 146.82% higher than conventional and nonimmersive VR, respectively. The diagnostic accuracies provided by the two cardiologists for each participant did not statistically differ from each other (*t*=–1.01, *P*=.31). The full-immersive VR was ranked as the most preferred display for performing group CHD discussions by 68% of the participants.

**Conclusions:**

The most preferred display system among medical trainees for visualizing medical images during group diagnostic discussions is full-immersive VR, with a trend toward improved diagnostic accuracy in complex anatomical abnormalities. Immersion is a crucial feature of displays of medical images for diagnostic accuracy in collaborative discussions.

## Introduction

Congenital heart disease (CHD) is the most common birth defect, occurring in 8/1000 neonates [1]. Management of CHD depends largely on anatomy [2], making detailed cardiac imaging (eg, echocardiogram and cardiac magnetic resonance imaging [MRI]) a necessity for accurate detection and preoperative planning of CHD. For preoperative planning of CHD, group multidisciplinary meetings are held between pediatric cardiologists, pediatric cardiac intensivists, and cardiac surgeons with cardiac imaging displayed in a conference-style room for review and discussion [3,4]. Cardiac imaging is typically displayed with visualization software geared toward Digital Imaging and Communications in Medicine (DICOM) formats, across a screen projector as either 2-dimensional (2D) images, cross-sections of 3-dimensional (3D) scans, or 3D volume renderings [5]. Despite the advancements in interactive 3D displays, the interpretation of cardiac imaging often relies on individual physicians to use 2D images and mentally reconstructing 3D objects.

Advances in medical imaging and additive technologies now allow for 3D printing of CHD anatomies [6]. 3D printing can use a variety of materials and colors to build customized and personalized anatomical models [7,8]. The printed models are useful for preoperative planning of CHD repair [6] as well as medical and surgical training [9-11]. However, 3D printing is cost- and time-intensive [7,12,13] and physically constraining, making a free-form visualization such as magnification or cropping challenging.

Virtual reality (VR) is an alternative 3D displaying modality with relatively lower costs and time use that provides free-form visualization. Although the physical models do not exist, realism is boosted through simulated physics [14,15] and implemented tools to deliver touch, auditory, and olfactory senses [16-18]. These attributes make VR one of the popular methods for training medical professionals [19-22], planning surgeries [23-26], and delivering therapies and rehabilitation [27-29]. Several commercial VR software programs are available for clinical decision making and surgical planning. Surgical Theater (Surgical Theater Inc) provides a platform allowing surgeons to virtually walk inside the patient anatomy to analyze neurological conditions and plan surgeries accordingly. Anatomy Viewer (The Body VR) converts DICOM images into 3D volume models that can be scaled, rotated, and cropped for identifying tumors and lesions. ImmersiveView Surgical Planning (ImmersiveTouch Inc) uses tactile haptic feedback and medical images for surgeons to visualize and rehearse surgeries. These commercial VR software programs all have functionality to visualize DICOM formatted data in 3D with multiple features assisting the diagnosing and surgical planning process.

Despite the advancements of VR in medicine, VR has been receiving criticism on its ability to facilitate collaboration, and the efficacy of VR has not been evaluated in group-based collaborative medical discussions, which is the bedrock of the clinical profession. VR necessitates full immersion for users to have bolstered sensation of the real world in VR [30]. However, full immersion also removes the face-to-face communication that contributes significantly to team productivity [31], moderation of team empowerment [32], knowledge transfer [33], and promotion of innovative solutions [34,35]. With limited knowledge existing on the influence of VR in collaboration, current VR development for medicine lacks emphasis on multiuser collaborative environments. Additional interaction features are essential for users to collaborate in VR. Furthermore, the multiuser environment needs to be optimized to balance network needs and avoid frame rate losses or lag. We developed a novel cardiac display software, Cardiac Review 3D, to address these shortcomings with the following design goals:

- Interactive display of medical anatomy: provide features to easily scrutinize the abnormalities of anatomies
- Knowledge sharing: enable storage of the virtual notes taken during the discussion for future access
- View sharing: establish an environment where multiple users can view the 3D medical images and provide feedback concurrently
- User experience: optimize the network and frame rates for a smooth user experience

Cardiac Review 3D was built with two levels of immersion. Full immersion is accomplished by using a head-mounted display (HMD), and nonimmersive VR uses a tablet. A conventional 2D display and the two extensions of Cardiac Review 3D were compared to identify the best display system for collaborative medical discussions. We hypothesized that VR, regardless of the level of immersion, better conveys the anatomical abnormalities of CHDs, bolstering diagnostic accuracy compared with the conventional display. This study was designed to imitate cardiac group diagnostic meetings where one physician controls the display systems presented to multiple medical providers who collaboratively identify the cardiac conditions related to CHD. Additionally, the study explored individual preferences of the display systems for group discussion.

## Methods

### Recruitment

This study was conducted under institutional review board approval. Medical trainees from Children’s National Hospital in Washington, DC, were recruited for the study (N=22). Of the participants, there were 1 first-year, 10 second-year, 4 third-year, and 1 fourth-year residents and 6 medical students. The participants were split into groups of 4 or 5 to maintain small group discussions. All participants gave informed consent prior to their participation. A minimum of 20 participants were recruited to achieve a power of 80% and a level of significance of 5% (2-sided) for detecting an effect size of 0.7 between pairs.

### Moderator

An experienced pediatric cardiologist from Children’s National Hospital acted as a moderator in the study. The moderator’s role and responsibilities were to give lectures on three chosen cases of CHD, provide instructions on how to interact with the display systems, and present answers to the diagnostic questions. The moderator’s interaction with the participants strictly followed a prewritten script.

### Medical Image Selection and Acquisition

#### Selection of Congenital Heart Disease Cases

The discussion topics included three cases of CHD: atrial septal defect (ASD), coarctation of aorta (CoA), and tetralogy of Fallot with pulmonary atresia and major aortopulmonary collateral artery (MAPCA). The selected cases each entail a spectrum of CHD in terms of surgical complexity and perioperative mortality risk, established by the Society of Thoracic Surgeons–European Association for Cardio-Thoracic Surgery’s STS-EACTS Congential Heart Surgery Mortality Categories (STAT Mortality Categories) [36]. Under the STAT Category, procedures are grouped from 1 to 5 (lowest to highest) based on estimated mortality risk and surgical difficulty. Under this classification, ASDs are classified under STAT Category 1 (estimated mortality risk of 0.3%), extended end-to-end repair of CoA is under STAT Category 2 (estimated mortality risk of 1.7%), and MAPCA is under STAT Category 4 (estimated mortality risk of 10.2%) [36].

Each case of CHD requires complex cardiovascular imaging for accurate diagnosis. ASDs, one of the most common forms of CHD, are typically well recognized on 2D echocardiography [37]. CoA, a discrete obstruction across the aortic isthmus, can also be identified by echocardiography; however, visualization of complex arch configurations (particularly after surgical repair) benefit from cross-sectional imaging such as cardiac MRI [38]. MAPCA is a very specific form of cyanotic CHD that results in loss of the pulmonary vessels, which are now directly connected to the aorta. Diagnostic imaging of MAPCA has been traditionally challenging and currently serves as a prime application for use of 3D imaging and 3D printing in cardiac surgical planning [39]. The mental workflow required for analysis of these defects is also intended to correlate with diagnostic complexity, as demonstrated in Table 1.

**Table 1:** Mental workflow for diagnosing three cases of congenital heart disease.

CHD^a^ case	Designed tasks	Mental workflow
ASD^b^	Recognition of primum-type ASD vs secundum-type ASD	Recognize atrial septal defect. Recognize location of atrioventricular valves. Identify primum ASD that is immediately superior to atrioventricular valves OR secundum ASD that is central to atrial septum.
CoA^c^	Recognition of unrepaired CoA vs repaired CoA with gothic arch	Recognize aortic arch regions: ascending aorta, transverse arch, descending aorta. Recognize normal head vessel anatomy and area immediately distal to left subclavian artery (aortic isthmus). Identify the pathological narrowing of aortic isthmus as CoA OR identify gothic arch shape in repaired CoA, which has a larger height to transverse ratio (taller height than width).
MAPCA^d^	Identify number of aortopulmonary collaterals and their respective takeoff points	Recognize aortic arch regions: ascending aorta, transverse arch, descending aorta. Recognize normal head vessel anatomy. Identify pathological aortopulmonary collateral. Identify the origin of each respective aortopulmonary collateral with respect to arch region throughout the heart.

^a^CHD: congenital heart disease.

^b^ASD: atrial septal defect.

^c^CoA: coarctation of aorta.

^d^MAPCA: major aortopulmonary collateral artery.

#### Medical Image Acquisition

**Figure 1:**
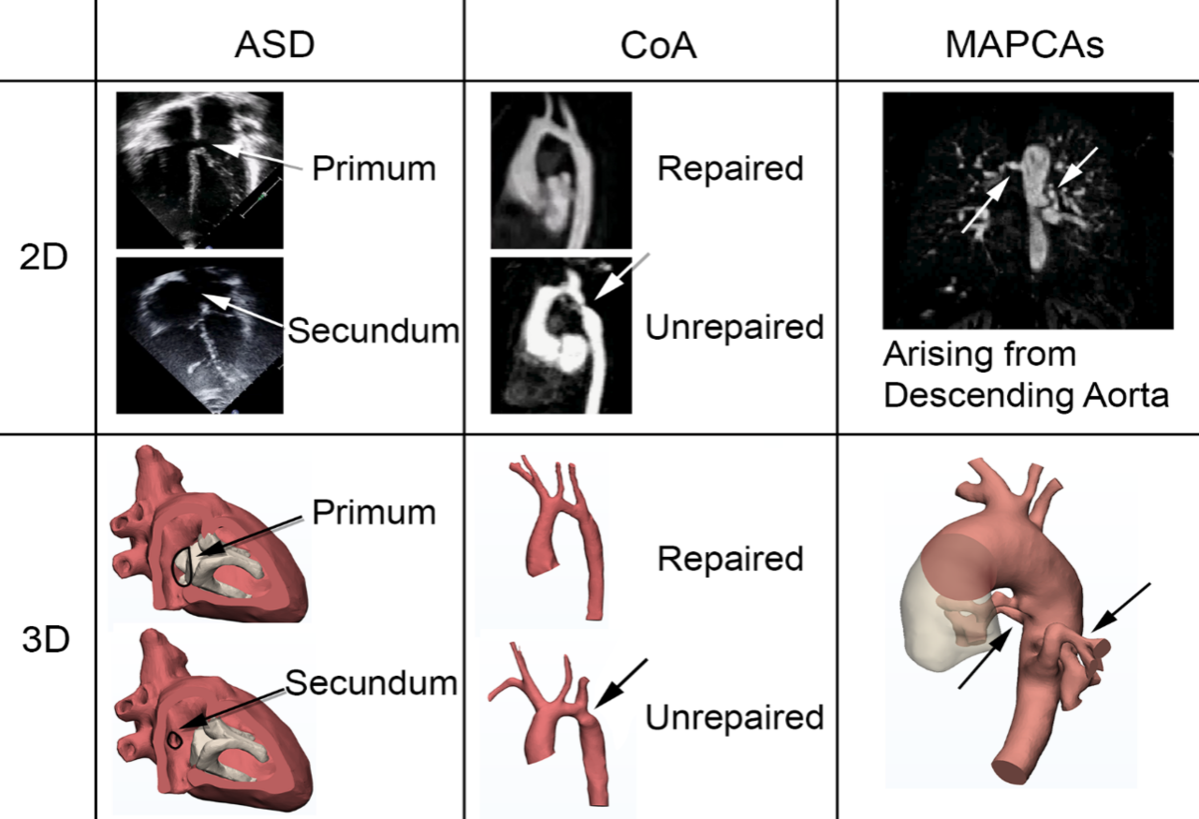
Medical images of the congenital heart disease cases: 2D (top) and 3D (bottom). Arrows represent the anatomical regions to scrutinize for correct diagnosis. ASD: atrial septal defect; CoA: coarctation of aorta; MAPCA: major aortopulmonary collateral artery.

Imaging datasets, acquired by standard-of-care imaging methods (MRI), were anonymized and exported as DICOM files. The DICOM files were manually segmented using thresholding and semiautomatic edge detection segmentation techniques in Mimics (Materialise) to create a 3D model, which was exported as a stereolithography file (see bottom 3D row in Figure 1) to be loaded into the Cardiac Review 3D software for group display.

### Medical Image Display Systems

**Figure 2:**
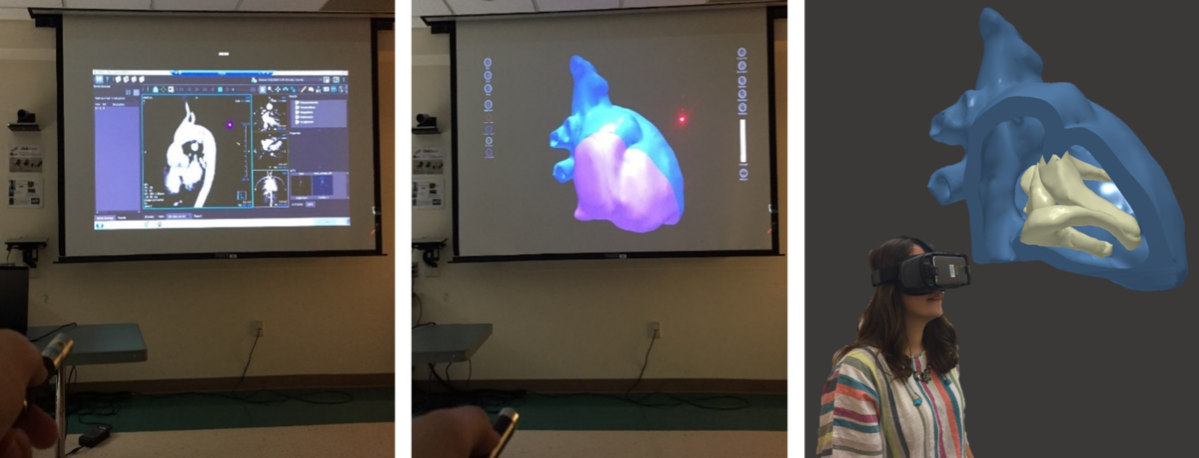
The setup of the conventional (left), nonimmersive virtual reality (middle), and full-immersive virtual reality (right) display systems in the study.

The study evaluated three medical imaging display systems—conventional, nonimmersive VR, and full-immersive VR (Figure 2)—for group diagnostic discussions of the CHDs. The conventional display system visualized 2D medical images on a projector screen. The nonimmersive VR system projected 3D medical images visualized in Surface Pro tablet (Microsoft Corp) onto a shared screen. A mobile HMD, Gear VR (Samsung Electronics Co Ltd), was provided to each participant for the full-immersive VR system, where 3D medical images were visualized in a virtual world.

#### Conventional Display System

**Figure 3:**
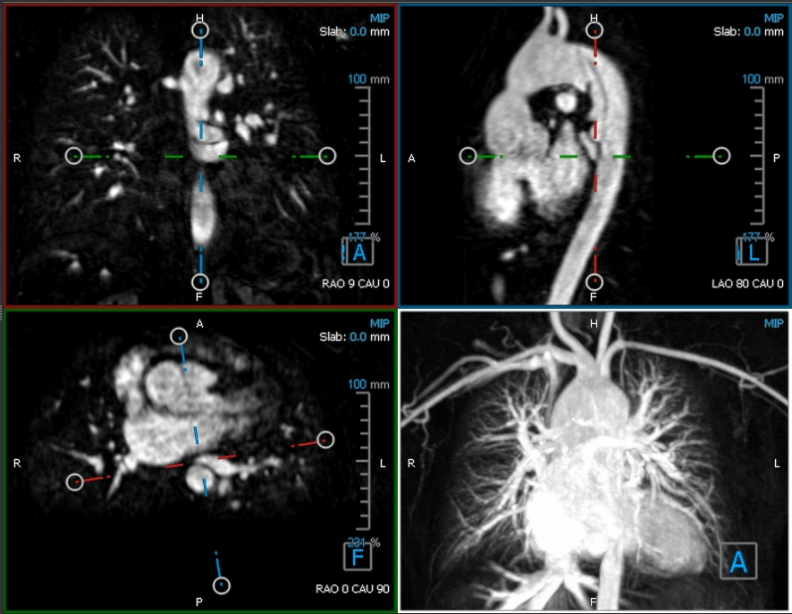
2D cross-sectional images of the repaired coarctation of aorta.

The conventional display system (CDS) used commercial cardiovascular imaging software running on a laptop that was duplicated on a projection screen (49×87 inch) located in front of the participants. For echocardiographic visualization of ASD, the 2D echo image of a standard 4-chamber apical view was exported from Xcelera (Philips Healthcare) in AVI format and presented via PowerPoint (Microsoft Corp) as a looped video. Cardiac MRI visualization of CoA and MAPCA was performed directly via Medis Suite MR (Medis Medical Imaging). Specifically, the 3D View suite was used to visualize cross-sectional anatomy through multiplanar reformatting technique, providing 2D cross-sectional images of the cardiac anatomy (Figure 3).

Each participant was provided with a unique color of laser pointer to pinpoint the images from a distance during the discussion phase using the CDS. While the discussion was in progress, the moderator was only responsible for complying with participants’ verbal directions for translating and rotating the 2D multiplanar reformatting view. The moderator did not provide any guidance toward the designated task.

#### Nonimmersive Virtual Reality Display System

Cardiac Review 3D was developed with the Unity engine (Unity Technologies) based on the four design goals: medical features, knowledge sharing, view sharing, and user experience. For medical features, a multitouch gesture interface with one finger to rotate, a 2-finger pinch gesture to zoom, and a 2-finger touch-and-drag gesture to pan were implemented. When loading multiple 3D models concurrently, each model was assigned a different color to ease the differentiation process. Interior (or back) faces of the 3D models were rendered with a desaturated color relative to exterior faces to accentuate the differences between inner and outer surfaces when clipping into the 3D model.

Knowledge sharing was accomplished through cloud-based storage of the cardiac datasets and associated annotated reports. The reports incorporated text labeling of 3D surface points, linear measurements, screenshots, and general annotations. Considering potential difficulty in estimating the true size of cardiac anatomies, two 3D marking points could be placed onto the 3D models to measure the lengths. The software provided options to export all markups and screenshots from the discussion into a PDF file to facilitate future review as well as a custom project file export to enable editing of 3D models and associated markups.

A tablet was chosen as the nonimmersive virtual reality display system (NIV) platform to support portability in surgical conferences, operating rooms, and intensive care unit settings. The view sharing of the tablet was achieved by a projection screen with laser pointers (Figure 2) and verbal requests for manipulating the anatomies, mimicking the CDS setup. Again, the moderator was only responsible for manipulating the 3D models as requested by the participants.

#### Full-Immersive Virtual Reality Display System

**Figure 4:**
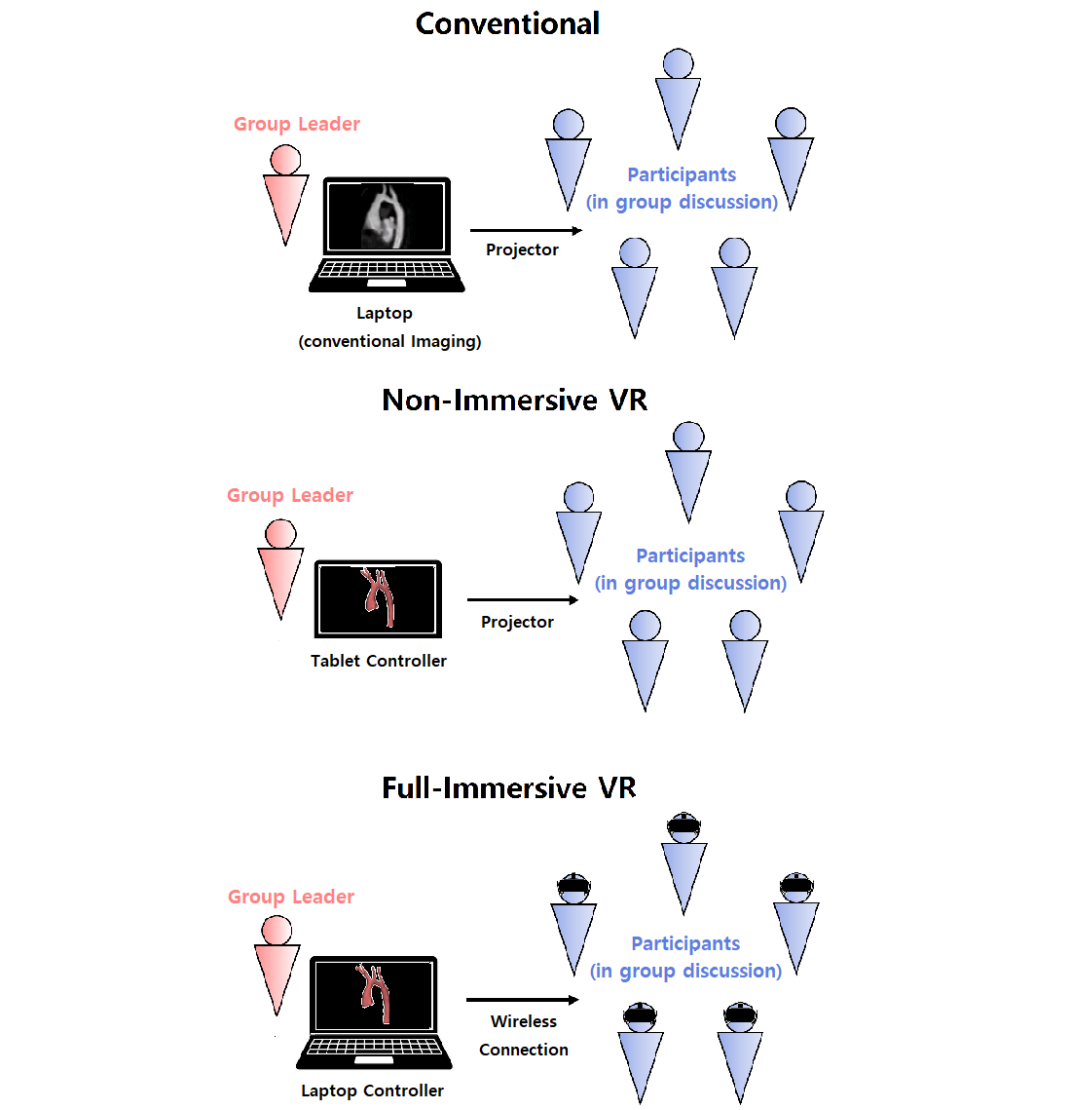
Diagram of group discussion format for each display system (from the top: conventional, nonimmersive virtual reality, and full-immersive virtual reality displays). VR: virtual reality.

The full-immersive virtual reality display system (FIV) incorporated the same medical review and knowledge-sharing features as the tablet platform of the Cardiac Review 3D (Figure 4). Loading and manipulating the 3D models, including zoom, rotation, and clipping, was controlled for all users by the moderator using a laptop running the Cardiac Review 3D in a server mode. The same interaction level and method were required between the participants and the moderator as the CDS and NIV. However, the view-sharing approach (ie, laser pointer) needed modifications since HMDs were worn by all patients, obstructing face-to-face communication. The server laptop and HMDs were connected via Wi-Fi to a wireless router to form a local network. Then the user datagram protocol was implemented to facilitate the network data transfer used to synchronize the 3D model manipulations from the laptop server to each client HMD.

This allowed for each HMD user to freely turn their heads to look around without translational components and to place a virtual pointer on the 3D surface model during the discussion, with their view of the virtual environment updating at 60 frames per second. For easy distinction, a unique pointer color was assigned to each HMD. The virtual pointer could be dropped anywhere on the 3D surface model by tapping on the touchpad located on the right side of the headset device. Selecting the location of this pointer placement was achieved by using the built-in gyroscope sensor of the Gear VR to determine the gaze vector of the users’ view relative to the 3D surface model at the time the touchpad was tapped. The gaze vector was then tested against the 3D surface to determine if the vector intersected the surface, and if so, the users’ unique colored virtual pointer was placed at that point by the software. The pointers placed by a participant were visible by all participants, facilitating discussion around specific 3D features visible on the inner or outer surfaces of the 3D models.

### Experimental Tasks and Procedure

A group orientation was provided at the beginning of the study. After the participants gave informed consent, the demographic survey was distributed and the moderator introduced himself and requested that participants greet each other and introduce themselves. The study was organized into 3 cases of CHD, each with 3 separate phases: lecture, group discussion, and postdiscussion survey. To avoid order bias, the 3 cases of CHD were randomly coupled with 3 display systems and provided in random order (Table 2). These selections were accomplished by running a MATLAB (The MathWorks Inc) script that generated random discussion combinations and orders.

**Table 2:** Discussion orders (congenital heart disease variation; display system) of each group.

Group	Discussion 1	Discussion 2	Discussion 3
1	CoA^a^; NIV^b^	MAPCA^c^; FIV^d^	ASD^e^; CDS^f^
2	ASD; FIV	CoA; CDS	MAPCA; NIV
3	ASD; NIV	CoA; FIV	MAPCA; CDS
4	MAPCA; CDS	ASD; FIV	CoA; NIV
5	CoA; CDS	MAPCA; FIV	ASD; NIV

^a^CoA: coarctation of aorta.

^b^NIV: nonimmersive virtual reality display system.

^c^MAPCA: major aortopulmonary collateral artery.

^d^FIV: full-immersive virtual reality display system.

^e^ASD: atrial septal defect.

^f^CDS: conventional display system.

For the lecture phase, the moderator prepared PowerPoint slides with a brief summary of each CHD case. The moderator explained the deviations from the norm presented for each CHD case under discussion and its standard diagnostic approach. The group discussion phase was solely held by the participants, who were not permitted to ask the moderator any questions that could be a hint at the CHD diagnosis. Discussions were limited to 10 minutes but could adjourn early if consensus were made within a group. All discussions were audio recorded for measuring the time duration for each group to reach a consensus.

The postdiscussion survey was provided to be answered individually, based on the possibility of individual learning variance from group discussions [40]. The discussion that used FIV included an additional survey about the experience of wearing the HMD. A comparison survey was given to each participant at the end of the study.

### Survey Design

#### Demographic Survey

The demographic survey consisted of 5 questions regarding the participant’s gender, year in residency, prior experience in VR, and impression of VR. Those who reported having prior experience in VR were requested to list the specific VR applications they tried. All participants were asked to score their impression of VR, which was categorized into negative, neutral, and positive. The strength of the negative and positive impression of VR was noted by increase in the magnitude of the value, from 1 to 5.

#### Postdiscussion Survey

The post discussion survey consisted of the diagnostics and peer assessment questionnaires. The diagnostics questionnaires were designed to measure the accuracy of the diagnosis made for each case of CHD using the different display systems. The ASD diagnostics questionnaire inquired about identifying the primum and secundum type of ASD. The CoA questionnaire prompted the participant to distinguish between the normal versus gothic arch. The MAPCA diagnostic questionnaire asked about identifying the number of MAPCAs and the respective origins of each MAPCA at the aortic arch. To further evaluate the confidence and depth of the diagnostics, participants were requested to back their statements with explanations. These responses were graded by two experienced cardiologists from Children’s National Hospital with a predetermined answer rubric after the completion of the study (Table 3). The grading was performed individually.

**Table 3:** Grading rubric of the diagnostic questionnaires used in the study.

CHD^a^	Answer (max +2)	Explanation (max +2)
ASD^b^	Case 1 is secundum ASD and case 2 is primum ASD (+2)	ASD in case 1 is central to atrial septum OR ASD in case 2 is above the atrioventricular valve (+2) If atrial septum or atrioventricular valves not specifically mentioned, only partial credit (+1)
CoA^c^	Arch 1 is gothic arch and arch 2 is normal arch (+2)	Arch 1 has larger height-to-transverse ratio (taller height than width) OR arch 2 has narrowing distal to the left subclavian artery (+2) If height-to-transverse ratio (taller height than width) OR narrowing distal to left subclavian/narrowing of isthmus not specifically mentioned, only partial credit (+ 1)
MAPCA^d^	Total = 4 MAPCAs	Two from transverse arch (+1); only partial credit if transverse arch not specifically named (+0.5) Two from descending aorta (+1); only partial credit if descending aorta not specifically named (+0.5)

^a^CHD: congenital heart disease.

^b^ASD: atrial septal defect.

^c^CoA: coarctation of aorta.

^d^MAPCA: major aortopulmonary collateral artery.

The peer assessment was provided for evaluating the ease of collaboration with their peers as a result of the display system used. The questionnaires included Q1: organization of the meeting; Q2: concentration; Q3: listening attentiveness; Q4: individual participation; Q5: knowledge exchange; Q6: perceived emotion; and Q7: perceived boredom [32-34]. All questionnaires were formatted into a 5-point Likert scale.

#### Comparison Survey

The comparison survey included the participants’ preferences and perspectives on the ease of the display systems for performing group diagnostic discussion. Both components were measured using a ranking system with 1 being the most preferred or easiest use and 3 being the least preferred and most difficult use of display system in group diagnostic discussions. The reasons and thought processes behind the ranking choices were noted.

#### Virtual Reality Usability Survey

The VR usability survey prompted participants to report any physical discomfort or motion sickness experienced when wearing the HMD. Eyeglass wearers were asked whether they wore their glasses with the HMD or took them off; they were also asked about their visual experience and physical comfort level regarding eyeglass and HMD interaction.

### Statistical Analysis

Kruskal-Wallis and Dunn tests were performed to confirm the level of medical experience matched between the assigned groups (n=5). Additionally, an analysis of variance test was performed to test whether diagnostic accuracies varied between the groups. The Kruskal-Wallis test followed by the Dunn test were also used in comparing the changes in diagnostic accuracy between the display systems in each CHD variation. Friedman and Wilcoxon signed-rank tests were used in peer assessment and the comparison survey to determine whether responses differed between display systems. A Fisher exact test was performed to determine the influence of usability of HMD (ie, motion sickness and physical discomfort) on the postdiscussion survey and comparison survey responses. The Kruskal-Wallis test was performed to explore any influence of impression of VR before experiment on preference rating. All statistical analysis was performed on R 64-bit version 3.5.3 software (R Foundation for Statistical Computing) with significance being .05 or lower in *P* values.

## Results

### Demographic Survey

Two participants claimed to have prior experience with using immersive VR from playing VR games and exploring real estate property in VR. Both participants had moderately positive impressions of VR (ie, 1 and 2 points on a scale between –5 and +5) compared with the group of 20 participants without prior experience with VR (2.6 [SD 1.96]). The Kruskal-Wallis test showed that the results on VR impression did not show any difference in the choice of display preference (CDS χ^2^=4.1, *P*=.53; NIV χ^2^=4.5, *P*=.49; FIV χ^2^=1.3, *P*=.93). Due to the small sample size of those with prior VR experience, we could not directly test if VR experience influenced the choice of the most preferred display.

### Group Assignment

Between 4 to 5 participants were assigned to each of 5 groups according to participant availability. The Kruskal-Wallis test (χ^2^_4_=10.7, *P*=.03) was used to gauge differences in medical experience levels between the groups (Table 4). Further investigation with the Dunn test using the Benjamini-Hochberg method showed that group 3 had more medically experienced participants than group 5 (*z*=–2.19, *P*=.048). However, the diagnostic accuracy performance in CHD cases between the 5 groups did not show any statistical difference based on analysis of variance.

**Table 4:** Dunn test results comparing participants’ years of medical experience between the groups.

Years of medical experience	Groups Z-score (*P* value)				
	1	2	3	4	5
1	N/A^a^	–2.32 (.05)	0.35 (.41)	–0.52 (.38)	–1.96 (.06)
2	–2.32 (.05)	N/A	2.54 (.06)	1.67 (.09)	0.23 (.41)
3	0.35 (.41)	2.54 (.06)	N/A	–0.82 (.30)	–2.19 (.048)
4	–0.52 (.38)	1.67 (.09)	–0.82 (.30)	N/A	–1.37 (.14)
5	–1.96 (.06)	0.23 (.41)	–2.19 (.048)	–1.37 (.14)	N/A

^a^N/A: not applicable.

### Discussion Time

The discussion times of each group (n=5) and CHD variation are shown in Table 5. Despite being classified as the least complex CHD case in the study, the averaged discussion times for ASD (172 seconds) were slightly longer than for CoA (159 seconds). The trend was visible in all groups except for group 3. All groups spent the longest discussion time on the MAPCA case. The averaged discussion time for MAPCA was 382 seconds, more than twice the time for the ASD and CoA discussions.

**Table 5:** Discussion times of the congenital heart disease variations for all groups and on average.

Discussion time	ASD^a^		CoA^b^		MAPCA^c^	
Group	Display system	Time (s)	Display system	Time (s)	Display system	Time (s)
1	CDS^d^	279	NIV^e^	262	FIV^f^	367
2	FIV	373	CDS	293	NIV	585
3	NIV	80	FIV	144	CDS	501
4	FIV	71	NIV	64	CDS	294
5	NIV	58	CDS	34	FIV	165
Average	N/A^g^	172	N/A	159	N/A	382

^a^ASD: atrial septal defect.

^b^CoA: coarctation of aorta.

^c^MAPCA: major aortopulmonary collateral artery.

^d^CDS: conventional display system.

^e^NIV: nonimmersive virtual reality display system.

^f^FIV: full-immersive virtual reality display system.

^g^N/A: not applicable.

### Postdiscussion Survey

**Figure 5:**
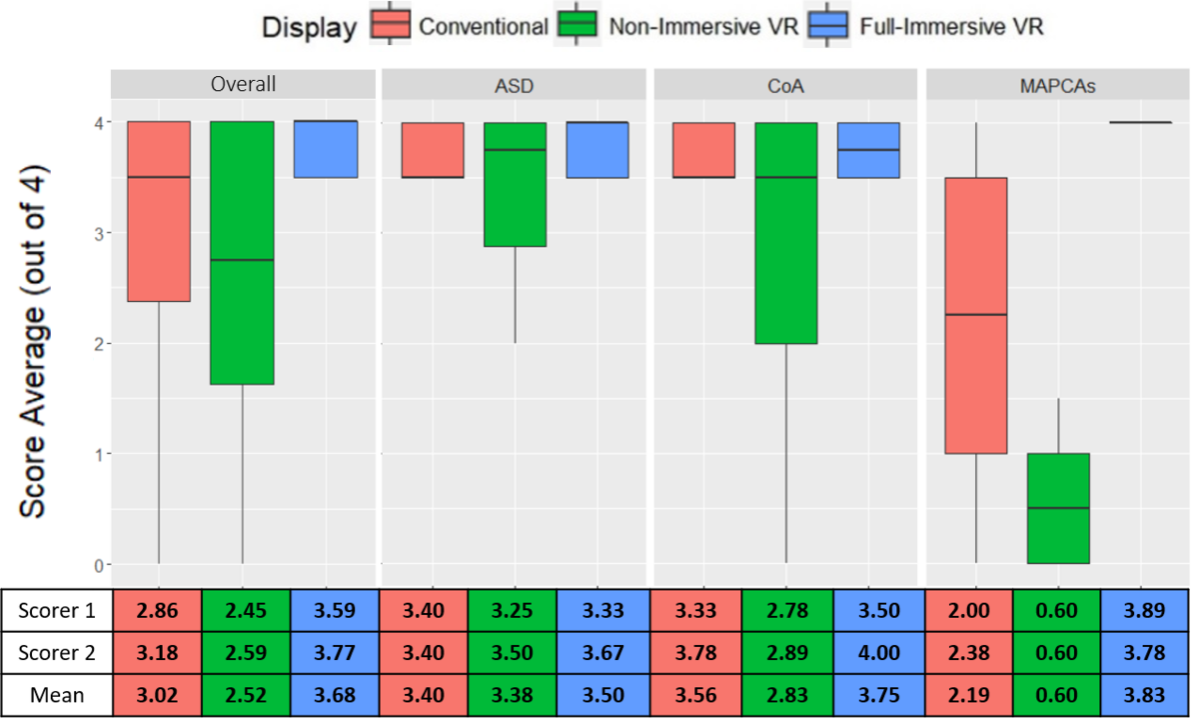
Diagnostic accuracy scores by type of congenital heart disease and display system (top) and by cardiologist (bottom). VR: virtual reality; ASD: atrial septal defect; CoA: coarctation of aorta; MAPCA: major aortopulmonary collateral artery.

Each participant received a diagnostic accuracy score ranging between 0 and 4 for each of the CHD cases. Two cardiologists individually graded the participants’ diagnostic performance according to the rubric (Table 3). The diagnostic accuracy scores were compared for each display system and CHD variation and broken down by cardiologist (Figure 5). No statistical differences were found between the grades of the two scorers (*t*=–1.01, *P*=.31).

The overall diagnostic accuracy difference between the display systems was statistically significant (χ^2^_2_=9.0, *P*=.01) where FIV had the highest averaged accuracy (Table 6). Differences became even more prominent with increasing case complexity (χ^2^_2_=14.1, *P*<.001; Table 6). For MAPCA, the average score percentage differences between the groups that used FIV to CDS and NIV were 54.49% and 146.82%, respectively. With the rise of CHD complexity, decreases in average scores of 35.59% and 82.86% were observed in CDS and NIV, respectively. Indeed, the Dunn test indicated that the averaged diagnostic accuracy of MAPCA for the FIV groups were significantly higher compared with the NIV groups (*z*=3.57, *P*=.001; Table 6).

**Table 6:** Kruskal-Wallis and Dunn test results on the overall congenital heart disease cases and broken down by type between the display systems on diagnostic accuracies.

Diagnostic accuracies	Kruskal-Wallis χ^2^ (*P* value)	Dunn Z-score (adj. *P* value)		
	Between display systems	CDS^a^ vs NIV^b^	CDS vs FIV^c^	NIV vs FIV
Overall	9.0 (.01)	0.65 (.51)	–2.21 (.04)	2.86 (.01)
ASD^d^	0.2 (.91)	–0.11 (.91)	–0.41 (>.99)	0.33 (>.99)
CoA^e^	1.0 (.62)	0.74 (.69)	–0.30 (.77)	0.88 (>.99)
MAPCA^f^	14.1 (<.001)	1.33 (.18)	–2.53 (.02)	3.57 (.001)

^a^CDS: conventional display system.

^b^NIV: nonimmersive virtual reality display system.

^c^FIV: full-immersive virtual reality display system.

^d^ASD: atrial septal defect.

^e^CoA: coarctation of aorta.

^f^MAPCA: major aortopulmonary collateral artery.

No significant difference between the display systems for each peer assessment questionnaire was found using the Friedman test (Q1 χ^2^=0.4, *P*=.82; Q2 χ^2^=3.4, *P*=.18; Q3 χ^2^=0.4, *P*=.82; Q4 χ^2^=1.3, *P*=.53; Q5 χ^2^=1.8, *P*=.42; Q6 χ^2^=0.2, *P*=.934; Q7 χ^2^=3.4, *P*=.19).

### Comparison Survey

Approximately two-thirds of the participants (15/22, 68%) ranked the FIV as the most preferred display system for performing group diagnostic discussions, and the rest of the participants (7/22, 33%) chose the NIV. The preference ranking ratings of the display systems were statistically significantly different based on the outcome of the Friedman test (χ^2^_2_=31.6, *P*<.001). Further testing with Wilcoxon signed-rank tests indicated that the median ranking rating of the FIV was statistically significantly higher than the median ranking rating of the NIV (*z*=3.33, *P*<.001) and CDS (*z*=4.10, *P*<.001). The NIV (10/22, 46%) and the FIV (8/22, 36%) received a similar number of votes for the easiest display system to use in group discussions or roughly twice as many as the number of the votes for CDS (4/22, 18%). The Friedman test revealed that a statistically significant difference between the display systems existed on the display system ease ranking (χ^2^_2_=20.6, *P*<.001). The median CDS ranking rating on ease of use were found to be statistically significantly lower than that of FIV (*z*=1.93, *P*=.047) and NIV (*z*=2.39, *P*=.01; Table 7) using the Wilcoxon signed-rank test.

**Table 7:** Friedman test and Wilcoxon signed-rank test results on the comparison survey between the display systems.

Comparison survey	Friedman test χ^2^ (*P* value)	Wilcoxon signed-rank test z-score (*P* value)		
	Between display systems	CDS^a^ vs NIV^b^	CDS vs FIV^c^	FIV vs FIV
Preference	31.5 (<.001)	3.33 (<.001)	4.11 (<.001)	1.83 (.049)
Easiness	20.6 (<.001)	2.39 (.01)	1.93 (.047)	–0.80 (.63)

^a^CDS: conventional display system.

^b^NIV: nonimmersive virtual reality display system.

^c^FIV: full-immersive virtual reality display system

### Virtual Reality Usability Survey

A total of 27% (6/22) of participant reported motion sickness from wearing the HMD. These participants were more likely to provide lower scores on concentration (*P*=.009) and knowledge exchange (*P*=.046) of the discussion using the FIV. Almost half (10/22, 45%) of participants experienced some level of physical discomfort especially around their noses from the heaviness of the HMD. Of the participants wearing glasses, 40% (2/5) removed them while using the HMD due to physical discomfort. However, no statistical differences were found between the groups that did or did not report physical discomfort and wore or did not wear glasses on all surveys (Table 8 and 9) and diagnostic performance using FIV (Table 8).

**Table 8:** Preference and ease of use ratings and diagnostic accuracy using the full-immersive virtual reality display system between the groups with and without physical discomfort, motion sickness, and eyeglass use.

Impact of usability on preference and diagnostic accuracies	Fisher exact test *P* value						
	Preference			Ease of use			FIV^a^ diagnostic accuracy
	CDS^b^	NIV^c^	FIV	CDS	NIV	FIV	Score
Physical discomfort	.57	.17	.85	.20	>.99	>.99	.77
Motion sickness	.15	.34	.43	.67	.29	.58	>.99
Glasses	>.99	>.99	>.99	.48	.51	>.99	>.99

^a^FIV: full-immersive virtual reality display system.

^b^CDS: conventional display system.

^c^NIV: nonimmersivevirtual reality display system.

**Table 9:** Peer assessment scores using the full-immersive virtual reality display system between the groups with and without physical discomfort, motion sickness, and eyeglass use.

Impact of usability on peer assessment	Fisher exact test *P* value						
	Q1	Q2	Q3	Q4	Q5	Q6	Q7
Physical discomfort	.57	.77	>.99	.55	.29	.48	.59
Motion sickness	>.99	.009	.07	.59	.046	.07	>.99
Glasses	>.99	.64	>.99	>.99	.54	>.99	.25

## Discussion

### Principal Findings

The FIV involved wearing HMD, which caused some physical discomfort and motion sickness and required training. These experiences could result in negative emotions, which are shown to be negatively related to team performances [41]. However, the FIV was rated as the most preferred medical image display system. The survey responses revealed that the realistic and interactive visualization ability such as interior viewing, rotating, and zooming in/out of the 3D anatomical models were the reasons for the better rating. CDS required succinct and accurate verbal directions for adjusting the sagittal, coronal, and frontal planes to orient the cross-sectional viewing and then processing them into the volumetric anatomy. The higher demand of mental conceptualization made the CDS the most difficult display system to use.

FIV has been facing criticism for the absence of face-to-face communication, which is related to reduced group collaboration quality [42-44]. Some studies emphasized the use of avatars as a remedy to improve social presence and communication in VR [45,46]. Although face-to-face communication was not featured in our clinical viewing software, providing shared perspective of anatomies and a mechanism for concurrent feedback was sufficient for enhanced group diagnostic performance. FIV showed strong diagnostic accuracy regardless of the CHD complexity unlike the other display systems, which showed worsened accuracy with increasing complexity.

### Limitations

To investigate the group diagnostic performances, an active discussion environment with experienced physicians across multiple disciplines was desirable. However, the recruitment process was challenged by a limited pool of trained physicians and their busy schedules. The study, therefore, recruited medical trainees who varied in years of medical experiences. A larger number of participants would increase the power of statistical results, but there was limited availability of pediatric residents (40 residents per class, with competing clinical demands), making 22 participants a realistic recruitment achievement. Due to the small data size, high standard deviations were observed in CDS and NIV, which increases the range of true diagnostic accuracy value. However, since the probability of false positive is lower with a smaller data sample size [47], the probability of falsely rejecting the differences in MAPCA diagnostic accuracy between display system is small. Nevertheless, we plan to narrow the spectrum of CHD cases down to the STAT categories of 4 and 5 in a future study to further evaluate the impact of displays on complex CHD cases.

Gender difference was disregarded in the study since the ratio of women to men was 19 to 3. Groups were formed based on participant availability. We confirmed through the Dunn test using the Benjamini-Hochberg method that except for groups 3 and 5 (*P*=.048), years of medical experience (eg, residency standing) did not vary between the groups. Since there was no statistical diagnostic performance difference found between the groups, we conclude that the participants’ years of experience were not an influential factor for performing tasks provided in the study.

The exact reason behind FIV being more preferred than the NIV could not be identified through the comparison survey results. Since the features of FIV and NIV are identical except for the immersion aspect, we suspect the perceived novelty of the VR experience could have impacted the choice in preferred displays. There were 20 participants who had never experienced VR prior to the experiments. To them, everything about FIV was novel, therefore they could have experienced increased perceived reward and individual preference toward the VR [48], leading them to prefer the FIV for CHD diagnosis tasks. To test the hypothesis, a future study will include a survey on novelty and compare its result on the preference rating.

The Cardiac Review 3D currently uses DICOM images that are 3D reconstructed and segmented into stereolithography file-formatted data. Since 3D reconstruction is not a routinely performed task in medicine, stereolithography is not stored in DICOM or part of the electronic patient records. To be compatible with the existing medical workflow, the logistics of storing 3D reconstructed data in DICOM is being identified.

### Conclusions

The Cardiac Review 3D is unique clinical viewing software with multiuser access and interaction. The software allows for visualization and manipulations of 3D anatomical models through zooming, rotating, panning, linear measurement, and adjustable clipping plane features. The text annotations, screenshots, and report features allow for taking notes in text and image forms for future access and archiving. User datagram protocol was implemented with virtual pointer for multiuser access and participation.

This study evaluated the group diagnostic discussion performances of the CDS, NIV, and FIV. Despite the lack of face-to-face communication and reduced concentration from motion sickness, the group discussions that used FIV demonstrated the best diagnostic accuracy overall and particularly for the most complex form of CHD. It also was the only display system that showed improving trend of diagnostic accuracy with increasing CHD complexity. The FIV relied on bulky hardware associated with physical discomfort, motion sickness, and increased learning process; however, it was still the most preferred display system for performing group diagnostic discussions.

The application of FIV has successfully supported improved diagnostic accuracy in CHD group discussions. FIV has the potential to bolster collaborative performance in discussion of other anatomies, medical education, and surgical planning. Expanding the significance of our findings, we believe that nonmedical fields such as computer-aided design, architecture, urban design, search and rescue, and military training that necessitate understanding of complex 3D structures may benefit from the use of FIV in collaborative discussions.

The Cardiac Review 3D provides features for medical doctors to visualize and interact with the patient anatomies in 3D in a group setting. Implementation of this technology could potentially bolster the diagnosis and preoperative planning of CHD, especially for complex cases (eg, MAPCA) by reducing the mental workload and capability of converting 2D cross-sectional images of anatomies into 3D and easily maneuvering around the anatomies.

## Abbreviations

2D: 2-dimensional
3D: 3-dimensional
ASD: atrial septal defect
CDS: conventional display system
CHD: congenital heart disease
CoA: coarctation of aorta
DICOM: Digital Imaging and Communications in Medicine
FIV: full-immersive virtual reality display system
HMD: head-mounted display
MAPCA: major aortopulmonary collateral artery
MRI: magnetic resonance imaging
NIV: nonimmersive virtual reality display system
STAT Mortality Categories: STS-EACTS Congential Heart Surgery Mortality Categories
STS-EACTS: Society of Thoracic Surgeons–European Association for Cardio-Thoracic Surgery
VR: virtual reality
